# Bisphenol Exposure Disrupts Cytoskeletal Organization and Development of Pre-Implantation Embryos

**DOI:** 10.3390/cells11203233

**Published:** 2022-10-14

**Authors:** Luhan Yang, Claudia Baumann, Rabindranath De La Fuente, Maria M. Viveiros

**Affiliations:** 1 Department of Physiology and Pharmacology, College of Veterinary Medicine, University of Georgia, Athens, GA 30602, USA; 2 Regenerative Biosciences Center (RBC), University of Georgia, Athens, GA 30602, USA

**Keywords:** preimplantation embryo, bisphenols, cytoskeleton, actomyosin, cell division

## Abstract

The endocrine disrupting activity of bisphenol compounds is well documented, but less is known regarding their impact on cell division and early embryo formation. Here, we tested the effects of acute in vitro exposure to bisphenol A (BPA) and its common substitute, bisphenol F (BPF), during critical stages of mouse pre-implantation embryo development, including the first mitotic division, cell polarization, as well as morula and blastocyst formation. Timing of initial cleavage was determined by live-cell imaging, while subsequent divisions, cytoskeletal organization and lineage marker labeling were assessed by high-resolution fluorescence microscopy. Our analysis reveals that brief culture with BPA or BPF impeded cell division and disrupted embryo development at all stages tested. Surprisingly, BPF was more detrimental to the early embryo than BPA. Notably, poor embryo development was associated with cytoskeletal disruptions of the actomyosin network, apical domain formation during cell polarization, actin ring zippering for embryo sealing and altered cell lineage marker profiles. These results underscore that bisphenols can disrupt cytoskeletal integrity and remodeling that is vital for early embryo development and raise concerns regarding the use of BPF as a ‘safe’ BPA substitute.

## 1. Introduction

Bisphenol compounds, such as bisphenol A (BPA), used in plastics manufacture are prevalent environmental toxicants. The widespread use of BPA in many common products has led to significant human exposure such that it is readily detected in blood, urine, and sweat [1]. In women, BPA has also been detected in the follicular fluid surrounding the oocyte in ovarian follicles, amniotic fluid during pregnancy and even breast milk in nursing mothers [2,3]. It is a recognized endocrine disruptor that can promote estrogen-like effects via activation of estrogen receptors [4,5]. Mounting evidence of the detrimental health effects posed by BPA exposure on multiple systems, including reproductive health [6,7,8], has led to restrictions on its use. As a result, there has been a marked increase in the utilization of chemically similar replacement analogues such as bisphenol F (BPF) and bisphenol S (BPS) [9,10,11]. Yet, more comprehensive analysis is needed to test the biological impact of these replacement compounds, particularly with regard to their effect on female gametes and embryos. Maternal exposure to BPA during early embryonic development (E0.5–3.5) in mice was shown to lower embryo implantation rates due to delays in embryo development and transport as well as reduced uterine receptivity [12,13]. Developmental toxicity of BPA, BPF and BPS has also been reported in zebrafish [14] and chick embryos [15]. Analysis of porcine embryos demonstrated that BPA can promote mitochondrial and DNA damage that impairs embryo development [16]. However, the underlying mechanisms by which bisphenols directly impact early embryo formation remains poorly understood.

Pre-implantation embryo development depends on precise timing and stability of early mitotic divisions as well as key transitions that involve critical remodeling of the cytoskeleton [17]. In mouse embryos, initial cleavage to the 2-cell stage typically occurs within 24 h post fertilization (hpf), and the embryo continues to divide symmetrically to the 8-cell stage. Dynamic morphological changes then ensue around the 8–16 cell stage, with blastomere (cell) polarization and compaction, characterized by a pronounced increase in cell–cell contacts [18]. The actomyosin network plays an important role in this process, which involves the establishment of tight and adherence junctions between blastomeres that will be essential for embryo sealing [19,20]. Cell polarization begins at the late 8-cell stage and is denoted by the formation of a unique apical domain in blastomeres located at the outer position of the embryo. This domain is characterized by F-actin recruitment to the cell cortex and expression of phosphorylated-Ezrin/Radixin/Moesin complex (pERM) that serves as a linker between the plasma membrane and underlying actin cytoskeletal network [21,22], emphasizing the critical role of cytoskeletal factors during embryo development [17]. Moreover, cell polarization at the 8–16 cell stage is associated with blastomere positioning to either an outer or inner location within the embryo, and the Hippo signaling pathway [23] that regulates cell specification of the inner cell mass (ICM) and trophectoderm (TE) progenitor cells [24,25]. The ICM cells are typically characterized by expression of the pluripotency marker NANOG, initially detected at the 16-cell stage, while TE cells express CDX2 [26,27]. Considering the critical role of cytoskeletal integrity and remodeling during key stages of embryo development [17], an increased understanding as to whether environmental toxicants, such bisphenols, disrupt cytoskeletal components in early development is warranted. Earlier studies reported microtubule and spindle disruptions in sea urchin eggs and embryos in response to BPA [28]. Moreover, we previously demonstrated that both BPA and BPF exert microtubule disruptive activity in female gametes that is highly detrimental to spindle organization and stability [29]. Thus, we sought to assess the effects of bisphenols during essential developmental transitions in the early embryo.

In this study, we tested the direct impact of bisphenols during in vitro mouse pre-implantation embryo development. The effects of acute BPA and BPF exposure were assessed at key stages, including 2-cell cleavage, cell polarization, morula and blastocyst formation. Experiments focused on potential bisphenol-mediated effects on mitotic division, the actomyosin network, cell polarization and cell lineage marker profiles. Our results reveal that even brief bisphenol exposure impedes cell division and disrupts cytoskeletal organization that is essential for these key developmental transitions. Our findings underscore that both BPA and BPF are highly detrimental to the developing embryo and provide novel insight regarding the underlying mechanisms of action.

## 2. Materials and Methods

### 2.1. Animals

For in vitro fertilization (IVF) studies, sperm and oocytes were obtained from B6D2F1 mice (C57BL/6J females × DBA/2J males). All mice were housed at a constant temperature (24–26 °C) and controlled light cycle (12 h light/dark), with food and water provided *ad libitum*. Animal use protocols were approved by the ‘Institutional Animal Care and Use Committee’ (IACUC) at the University of Georgia (Athens, GA, USA), and all experiments were conducted in accordance with the stipulated guidelines.

### 2.2. In Vitro Fertilization (IVF), Embryo Culture and Bisphenol Exposure

Pre-implantation embryos were generated by IVF using standard protocols in our laboratory [30]. To promote ovarian follicle development and ovulation, female mice were treated with 5 IU pregnant mare serum gonadotrophin (EMD Biosciences, San Diego, CA, USA) followed by 5 IU human Chorionic Gonadotrophin (EMD Biosciences) 44–48 h later. For IVF, cumulus-enclosed oocyte complexes (COCs) were recovered from the oviducts of 4–7 female mice from the same litter approximately 16 h post hCG treatment. The COCs were combined with sperm of a single male in micro-droplets of minimal essential medium (MEM) supplemented with 3 mg/mL bovine serum albumin (BSA, Sigma Aldrich, St. Louis, MO, USA). After a 4 h culture, the presumptive zygotes were rinsed three times in fresh media then transferred to 200 µL MEM micro-droplets under oil until 24 h post fertilization to undergo the first cleavage to the 2-cell stage. For subsequent embryo development, at 24 hpf the embryos were briefly rinsed and transferred to 200 µL KSOM media micro-droplets under oil and maintained up to 96 hpf at 37 °C under an atmosphere of 5% O_2_, 5% CO_2_ and 90% N_2_.

The effect of brief bisphenol exposure was tested at key stages of pre-implantation embryo development. Bisphenol A (BPA) and bisphenol F (BPF) were purchased from Sigma-Aldrich (St. Louis, MO, USA) and dissolved in Dimethyl Sulfoxide (DMSO, Sigma-Aldrich) for stock solution preparation. In different experiments, zygotes and embryos were generated by IVF, as described above, and were randomly allocated into either control or bisphenol treatment groups at specified times, including 4, 48, 53, or 72 h post fertilization (hpf) that correspond to the timing of the first cleavage, cell polarization, morula and blastocyst formation, respectively. A minimum of 3 replicates were conducted for each experiment, with approximately 30–40 zygotes/embryos per treatment group. In brief, the developing embryos were cultured in media supplemented with a low or high dose (5, 25 µg/mL) of either BPA or BPF, while control embryos were cultured in fresh media with 0.05% DMSO. The bisphenol concentrations used are similar to previous studies with oocytes [31,32] and embryos [16] as well as our studies demonstrating cytoskeletal disrupting activity [29]. 

### 2.3. Live Cell Imaging

Timing of the first mitotic cleavage division post fertilization was assessed by live cell imaging, under bright-field conditions. In vitro fertilization was conducted as described. At 4 hpf, the presumptive zygotes were rinsed in fresh media and transferred to a 200 µL MEM/BSA micro-droplet, overlaid with mineral oil, in a 30 mm glass bottom Petri dish that was placed within an environmental chamber (Tokai Hit, Fujinomiya, Shizuoka, Japan) for confocal imaging. The embryos were maintained at 37 °C, with an atmosphere of 5% CO_2_, 5% O_2_ and balance Nitrogen. Live cell imaging began at 5 hpf and the embryos were monitored at 20-min intervals for 24 h using a Nikon Eclipse Ti-U/D-Eclipse C1 laser scanning confocal microscope equipped with a 10× objective lens. Image acquisition was conducted using EZ-C1 software (Nikon, Melville, NY, USA) with a step size of 5 µm and a Z-stack of 100 µm. Imaging data were analyzed using NIS Elements software (Nikon).

### 2.4. Immunofluorescence

Embryos were fixed and immunolabeled with specific antibodies as previously described [33] to detect specific markers including, H3K9me3 (1:500, Cat#ab5819, Abcam, Cambridge, MA, USA), acetylated α-Tubulin (1:1000, Cat# T6793, Sigma Aldrich), NANOG (1:200, Cat#REC-RCAB002P-F, Cosmo Bio, Carlsbad, CA, USA), CDX2 (1:200, Cat#AM392GP, Biogenex, Fremont, CA, USA), myosin II chain B (1:1000, Cat#B268382, BioLegend, San Diego, CA, USA), phospho-Ezrin/Radixin/Moesin (1:200, Cat#3726S, Cell Signaling Technology, Beverly, MA, USA), or YAP1(1:1000, clone 2F12, Cat#H00010413-M01, Abnova, Taipei City, Taiwan). In brief, fixed embryos were incubated with specific primary antibodies overnight at 4 °C, then washed 3 times and incubated (1 h) at 37 °C with specific Alexa Fluor conjugated 488 or 555 secondary antibodies (1:1000, Cat#A11070, A21425, A11017, A21430, Life Technologies, Eugene, OR, USA) or Alexa Fluor 633 phalloidin (1:1000, Cat#A22284, Life Technologies, Waltham, MA, USA). After a final wash, the embryos were transferred onto glass slides and overlaid with mounting medium (Vecta Shield, Vector Laboratories, Burlingame, CA, USA) containing DAPI (4′,6-diamidino-2-phenylindole) to counterstain the DNA. Fluorescence was assessed using either a Leica DMRE upright fluorescent microscope (Leica Microsystem, Deerfield, IL, USA) or a Zeiss LSM 710 confocal microscope equipped with 40× and 63× objectives and imaging software. In some experiments the embryos were also assessed by super-resolution structured illumination microscopy (SR-SIM) using a Zeiss Elyra S1 system equipped with 100x oil immersion lens, and ZEN 2011 software with a SIM analysis module for image acquisition at the Biomedical Microscopy Core (BMC) facility, University of Georgia.

### 2.5. Statistical Analysis

Data are presented as either mean percentages (±s.e.m.) or median values from a minimum of three independent experimental replicates. Scoring was performed in a nonblinded manner. The GraphPad Prism software was used for data analysis and preparation of all graphs. Box and whiskers plots represent min. and max. values with the median shown by the central horizontal line, and box limits corresponding to the 25th and 75th percentiles. The data were analyzed by one-way ANOVA for multiple comparisons between groups, and differences were considered to be significant when *p* < 0.05.

## 3. Results

### 3.1. Bisphenol Exposure Shortly after Fertilization Disrupts the First Cleavage Division

**Figure 1:**
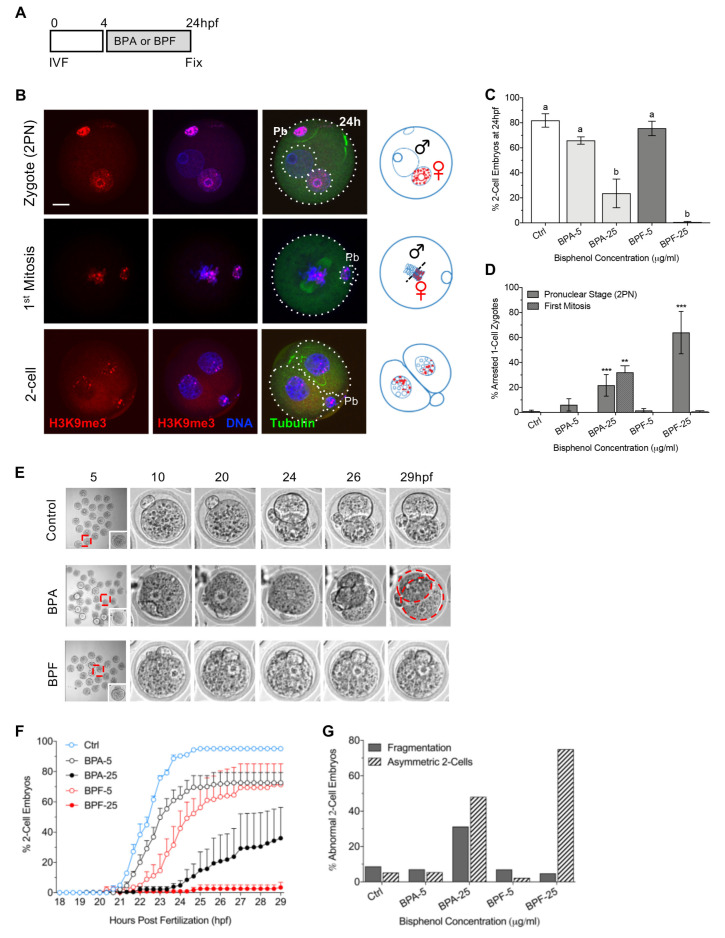
Brief exposure to BPA or BPF at the 1-cell zygote stage disrupts the first mitotic division. (**A**) Timing of BPA or BPF exposure (shown in grey) and embryo fixation. (**B**) Representative images of embryos fixed at 24 hpf and corresponding illustrations. H3K9me3 is shown in red, DAPI- labeled DNA in blue and acetylated α-tubulin in green. Pb: polar body. Scale bar of 20 µm. Percent (mean ± s.e.m.) total (**C**) 2-cell stage embryos or (**D**) arrested zygotes, at either the pronuclear stage or first mitosis. Data summary from 3–5 replicates, with a total of 100–150 embryos per group. (**E**) Representative images from live-cell imaging during 5–29 hpf. Red squares denote the inset. Dashed red lines denote asymmetric blastomeres. (**F**) Timing of 2-cell cleavage onset assessed at 20-min intervals. (**G**) Percent (mean ± s.e.m.) of 2-cell embryos that show disrupted cleavage and fragmentation. Data summary from 2–3 replicates, with a total of 80–180 embryos per group. hpf: hours post fertilization. Different letters denote significant differences (*p* < 0.05) between groups. ** *p* < 0.01, *** *p* < 0.001 compared to the control group.

To test the effect of bisphenol exposure during the first cleavage division, 1-cell zygotes were cultured in media with either BPA or BPF (5, 25 µg/mL) from 4 to 24 hpf then immediately fixed for immunofluorescence (Figure 1A). Fertilization was confirmed by differential chromosome complement labeling with the maternal-specific histone marker, H3K9me3 [34] and the number of 2-cell embryos determined. In addition, chromatin and microtubule configurations were used to classify arrested zygotes at either the pronuclear (PN) or first mitosis stages (Figure 1B–D). In the control group, approximately 81% of the embryos reached the 2-cell stage by 24 hpf. However, exposure to high dose (25 µg/mL) BPA or BPF significantly reduced the 2-cell rate to 25% and less than 1%, respectively (Figure 1C). The majority of arrested zygotes in the BPF group failed to develop beyond the PN stage, whereas BPA-exposed zygotes arrested at either the PN stage or the first mitosis (Figure 1D). Next, live-cell imaging was used to assess the timing of 2-cell cleavage (Figure 1E,F). In the control group, cleavage onset occurred at approximately 21 hpf, with 90% of the cleaved embryos reaching the 2-cell stage by 23 hpf. The blastomeres appeared similar in size with no overt morphological abnormalities (Figure 1E–G). Notably, bisphenol exposure significantly delayed the onset of 2-cell cleavage with high BPF almost fully blocking division. Moreover, the few cleaved embryos showed poor morphology with increased rates of asymmetric division and blastomere fragmentation (Figure 1E,G). These data demonstrate that brief BPA or BPF exposure shortly after fertilization promotes a significant delay or block of the first mitotic division. Interestingly, BPF exposure was more detrimental than BPA, leading to almost complete zygote arrest at the PN stage.

### 3.2. Transient Bisphenol Exposure at the Zygote Stage Disrupts the Cortical Actomyosin Network and Limits Subsequent Embryo Development

**Figure 2:**
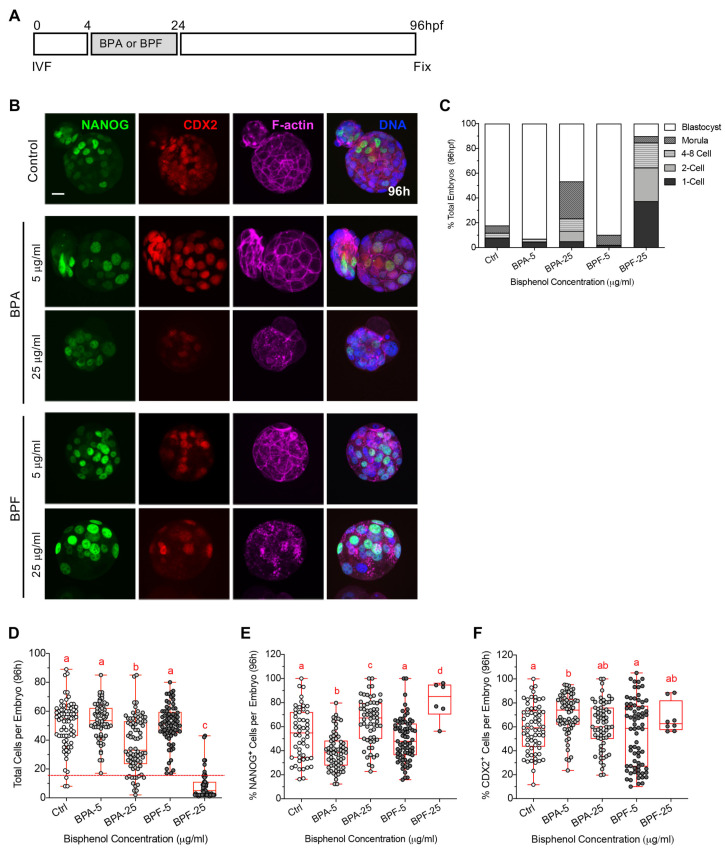
Transient bisphenol exposure at the 1-cell zygote stage impairs development to the blastocyst stage, despite compound removal. (**A**) Timing of BPA or BPF exposure (4–24 hpf, grey) and embryo fixation (96 hpf). (**B**) Representative images of embryos fixed at 96 hpf, labeled with anti-NANOG (green), anti-CDX2 (red) and Phalloidin to detect F-actin (magenta). DAPI-labeled DNA is shown in blue. Scale bar of 20 µm. (**C**) Percent (mean ± s.e.m.) total embryos at different stages of pre-implantation development at 96 hpf. (**D**) Total cells per embryo in each group. Percent (**E**) NANOG-positive and (**F**) CDX2-postive cells detected in embryos with more than 16 total cells. Box and whiskers plots represent the min and max values, with the center horizontal line representing the median. hpf: hours post fertilization. Different letters (red) denote significant differences (*p* < 0.05) between groups.

Next, we sought to assess the effects of transient BPA and BPF exposure during the first cleavage division on subsequent embryo development (Figure 2). To this end, zygotes were briefly exposed to BPA or BPF from 4 to 24 hpf, as previously described, then thoroughly washed and transferred to fresh KSOM media alone for culture until 96 hpf (Figure 2A), when embryo development was assessed in all groups (Figure 2B,C). The majority of embryos in the control and low dose (5 µg/mL) BPA or BPF groups reached the blastocyst stage by 96 hpf. In contrast, brief exposure to the higher concentration (25 µg/mL) of BPA or BPF significantly impaired embryo development, despite compound removal at 24 hpf. In the high dose BPA group, only 46% of the embryos reached the blastocyst stage, 30% were morula and 24% arrested with less than 4–8 cells. Notably, transient exposure to 25 µg/mL BPF was more detrimental, such that only 15% of the embryos developed to the either morula or blastocyst stage, 48% arrested with 2–8 cells and 37% never cleaved (Figure 2C).

To further assess the embryos, DAPI-labeled DNA was used to determine the total cell number and all embryos were assessed by immunofluorescence to detect NANOG and CDX2, key markers for the inner cell mass (ICM) and trophectoderm (TE) cell lineages, respectively (Figure 2B,E,F). Previous studies report only weak NANOG and CDX2 expression at the 8-cell stage. NANOG expression increases in 16-cell stage embryos, while CDX2 is gradually restricted in a subset of cells [35,36,37]. Therefore, embryos with less than 16-cells were excluded when comparing NANOG and CDX2-labeled cells in all groups. Embryos in the high dose (25 µg/mL) bisphenol groups contained significantly fewer total cells. Notably, only 15% of embryos in the BPF group contained 16 or more total cells (Figure 2D). Lineage marker profiles were also altered in response to bisphenol exposure (Figure 2E,F). In embryos transiently exposed to 5 µg/mL BPA, the percent NANOG positive cells was reduced while CDX2 positive cells increased. In contrast, the proportion of NANOG positive cells increased in embryos exposed to higher (25 µg/mL) BPA or BPF. These altered marker profiles, point to a disruption in embryo development and the regulation of cell lineage specification.

**Figure 3:**
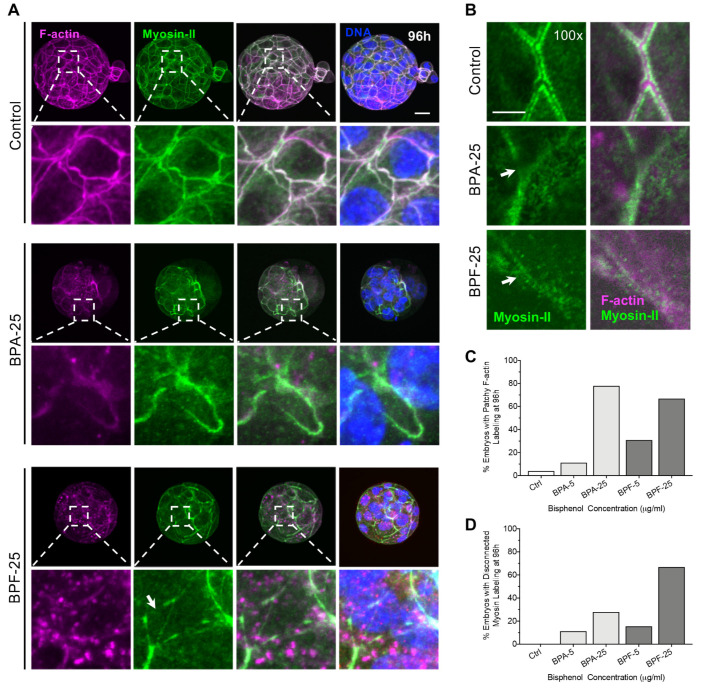
Embryos transiently exposed to bisphenol post fertilization show disruptions in the actomyosin network at 96 hpf. (**A**) Representative images of the actomyosin network in embryos fixed at 96 hpf labeled with anti-myosin II (green) and Phalloidin to detect F-actin (magenta). DAPI-labeled DNA is shown in blue. Dashed box and lines denote the zoomed region below. Arrowheads indicate disconnected myosin-II filaments. Scale bar of 20 µm (**B**) Optical sections of the actomyosin network at the cortex between blastomeres visualized at high (100×) magnification. Scale bar of 5 µm. Percentage of total embryos with (**C**) irregular, “patchy”, F-actin staining and (**D**) disconnected myosin-II filaments. Data summary of 2 replicates, with a total of 10–25 embryos per group.

Cytoskeletal components, such as actin microfilaments, play an important role in pre-implantation embryo development. For example, both cell division and compaction are highly dependent on the force generated by actomyosin contractility [38]. We, therefore, assessed F-actin and Myosin-II labeling in all embryos at 96 hpf (Figure 3). In control blastocysts, F-actin was detected at the cell cortex (Figure 3A). Higher magnification revealed clear myosin-II labeling along the actin filament, forming a well-defined tubular structure beneath the cell membrane (Figure 3B). In contrast, the actomyosin network was disrupted in BPA and BPF-treated embryos, with F-actin showing an irregular punctate and ‘patchy’ appearance together with weak and disconnected myosin-II filament labeling (Figure 3B–D). In sum, these data demonstrate that embryo development to the blastocyst stage is impaired by brief exposure to the bisphenols during the zygote stage, despite compound withdrawal at 24 hpf. Significantly fewer embryos reach the blastocyst stage. Moreover, these embryos show reduced total cell numbers, altered expression of key cell lineage markers and pronounced disruption of the actomyosin network.

### 3.3. Bisphenol Exposure during the 4–8 Cell Stage Disrupts Initial Cell Polarization and Division

**Figure 4:**
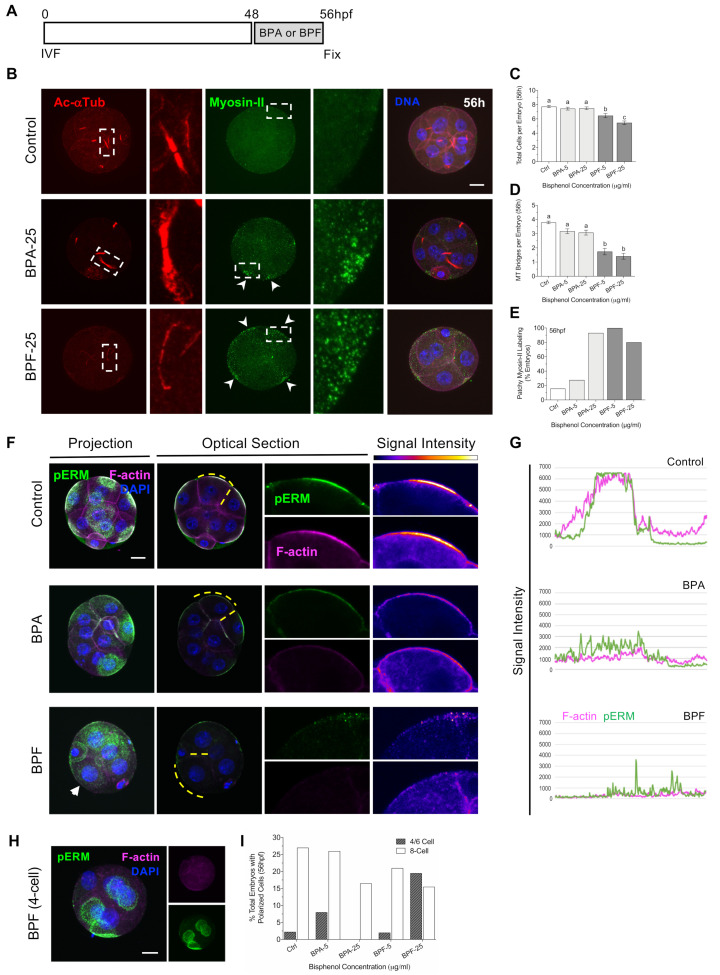
Brief bisphenol exposure disrupts cell division and polarization during 4 to 8-cell stage. (**A**) Timing of BPA or BPF (48–56 hpf, grey) exposure and embryo fixation. (**B**) Representative images of embryos fixed at 56 hpf and double-labeled with anti-myosin II (green) and anti-acetylated α-Tubulin (red) to detect microtubule (MT) bridges between blastomeres. DAPI-labeled DNA (blue). Dashed boxes indicate zoomed adjacent panels showing a 7.5x magnification. Arrow heads denote irregular, patchy, myosin-II staining. Total (mean ± s.e.m.) (**C**) cell number and (**D**) MT- bridges per embryo in all groups. (**E**) Percent (mean ± s.e.m.) of total embryos with irregular patchy myosin-II staining. Summary of data from 3–5 replicates with a total of 40–50 embryos in each group. Different letters denote significant differences (*p* < 0.05) between groups. (**F**) Representative images of the apical domain in polarized cells in embryos double-labeled with anti-phosphorylated-ERM (pERM, green) and F-actin/phalloidin (magenta). (**G**) Representative signal intensity plots for pERM (green) and F-actin (magenta) at the apical domain. (**H**) Representative image of a polarized 4-cell embryo. (**I**) Percent (mean ± s.e.m.) of total embryos with polarized cells in all groups. Scale bar of 20 µm. hpf: hours post fertilization.

Next, we assessed the effect of bisphenol exposure on cell division and the actomyosin network during successive stages of embryo development, beginning with the 4 to 8-cell stage when embryos undergo initial cell polarization. The actomyosin network plays a critical role in Par-aPKC complex recruitment to a unique apical domain that forms during polarization at the 8-cell stage [39] that, in turn, influences initial cell specification [40]. In brief, 4-cell embryos (at 48 h post fertilization) were cultured with media supplemented with BPA or BPF (5, 25 μg/mL) for 8 h before fixation at 56 hpf (Figure 4A). Total cells per embryo were determined as well as the number of microtubule (MT) bridges, labeled with acetylated tubulin, between blastomeres (Figure 4B–D) that denote cell division. Embryos briefly exposed to either 5 or 25 μg/mL BPF contained significantly fewer total cells and fewer MT bridges (Figure 4C,D) that appeared thinner compared to controls, indicating a possible block or delay in cell division. In contrast, there was no difference in total cells or the number of MT bridges per embryo in either BPA group. Yet, the MT bridge structure appeared thicker, suggestive of recent cell division in these embryos (Figure 4B). The majority of embryos exposed to BPF and high dose BPA also showed punctate myosin-II labeling at the cell cortex (Figure 4B,E), indicative of the cytokinesis cleavage furrow and likely recent/delayed cell division.

At the 8-cell stage, the outer cells of the embryo become polarized with the formation of a unique apical domain, denoted by a ‘cap-like’ labeling with the microvillar marker, pERM (phosphorylated Ezrin/Radixin/Moesin) and an F-actin ring at the cortex [40,41]. Therefore, we evaluated apical pERM and F-actin labeling in all groups (Figure 4F). In control 8-cell embryos, cortical enrichment of F-actin together with a bright pERM-labeled ‘cap’ was observed in the outer polarized blastomeres. In contrast, BPA and BPF-treated embryos contained a mixture of outer polarized and ‘apolar’ blastomeres with limited apical domain formation, indicating a disruption in initial cell polarization. Fluorescence analysis also revealed reduced signal intensity for both pERM and F-actin (Figure 4F,G). Surprisingly, some apical domain formation was observed in embryos that remained at the 4–6 cell stage in the BPF-treated group (Figure 4H,I). This indicates that while cell polarization typically occurs at the late 8-cell stage -this process is not strictly dependent on total blastomere number. These results demonstrate that bisphenol exposure during the 4–8 cell stage disrupts cell division and initial cell polarization, with reduced F-actin recruitment and apical domain formation, pointing to defects in cytoskeletal organization and/or integrity. 

### 3.4. Bisphenols Disrupt the Actin Ring “Zippering” Process during Morula Formation

**Figure 5:**
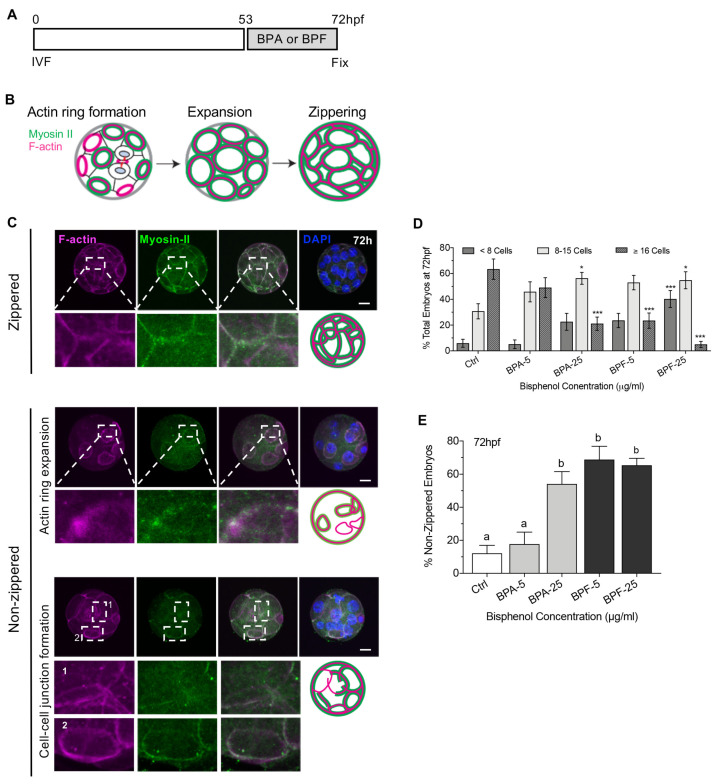
The process of actin-zippering for embryo sealing is disrupted by bisphenol exposure during compaction. (**A**) Timing of BPA or BPF exposure (53–72 h, grey) and embryo fixation. (**B**) Illustration of the zippering process between blastomeres, including cortical actin ring formation, expansion, and connection with neighboring rings (zippering) for embryo sealing. (**C**) Representative images of zippered and non-zippered embryos double-labeled with anti-myosin II (green) and Phalloidin/F-actin (magenta) at the cell junction between blastomeres. DAPI-labeled DNA is shown in blue. Scale bar of 20 µm. Dashed box and lines denote the zoom region below. (**D**) Percent (mean ± s.e.m.) of total embryos with less than 8 cells, 8–15, or 16-cells or more at 72 hpf. (**E**) Percent (mean ± s.e.m.) of total “zippered” embryos at 72 hpf. Summary data of 7–8 replicates with total 100–150 embryos in each group. Different letters denote significant differences (*p* < 0.05) between groups. * *p* < 0.05, *** *p* < 0.001 compared to the control group.

Apical domain formation and cell polarization play an important role in cell lineage specification as well as embryo sealing and compaction at the morula stage [20]. Therefore, we next tested the effect of bisphenol exposure during morula formation (Figure 5) at the 8–16 cell stage. As embryos develop to the morula stage, a pronounced increase in cell–cell adhesion and tight junctions creates an outer permeability layer to seal the embryo. Comprehensive studies [20] revealed that this sealing process involves formation of the unique cortical F-actin rings at the apical domain of the outer blastomeres as initially observed at the 8-cell stage. These F-actin rings then expand to contact neighboring rings on adjacent blastomeres and undergo a ‘zippering’ process to form tight junctions (Illustrated in Figure 5B). The established outer permeability layer is essential for subsequent expansion of the blastocyst cavity [20]. To test whether bisphenol exposure affects this critical process, at 53 hpf embryos were transferred to media supplemented with either BPA or BPF (5, 25 μg/mL) while the control group was cultured in fresh media alone and fixed at 72 hpf (Figure 5A). Total cells per embryo were determined by DAPI-labeled DNA, while actin-ring formation and ‘zippering’ at the cell cortex was assessed by F-actin and myosin-II labeling of the actomyosin network.

Embryos cultured with the high dose BPA or BPF contained significantly fewer total cells compared to controls. Notably, even exposure to lower dose (5 µg/mL) BPF was highly detrimental at this stage, with few embryos containing 16-cells (Figure 5C,D). Labeling of the actomyosin network (Figure 5C) revealed that almost 90% of control embryos were fully ‘zippered’, denoted by actin ring expansion and contact between rings on adjacent blastomeres, indicative of embryo sealing. In contrast, the percent of fully ‘zippered’ embryos was significantly lower following brief culture with high dose BPA or both doses of BPF (Figure 5E). The non-zippered embryos exhibited disconnected myosin-II filaments with reduced actin ring expansion or contact between adjacent blastomeres. These data demonstrate that a brief bisphenol exposure during morula formation impairs cell division leading to fewer total cells per embryo. Importantly, BPA and BPF exposure also disrupted the actomyosin network that plays an essential role in actin ring expansion and ‘zippering’ between blastomeres for embryo sealing. 

### 3.5. Bisphenol Exposure during Morula Formation Disrupts Cell Polarity, YAP1^+pos^ Cell Distribution and Lineage Marker Profiles

**Figure 6:**
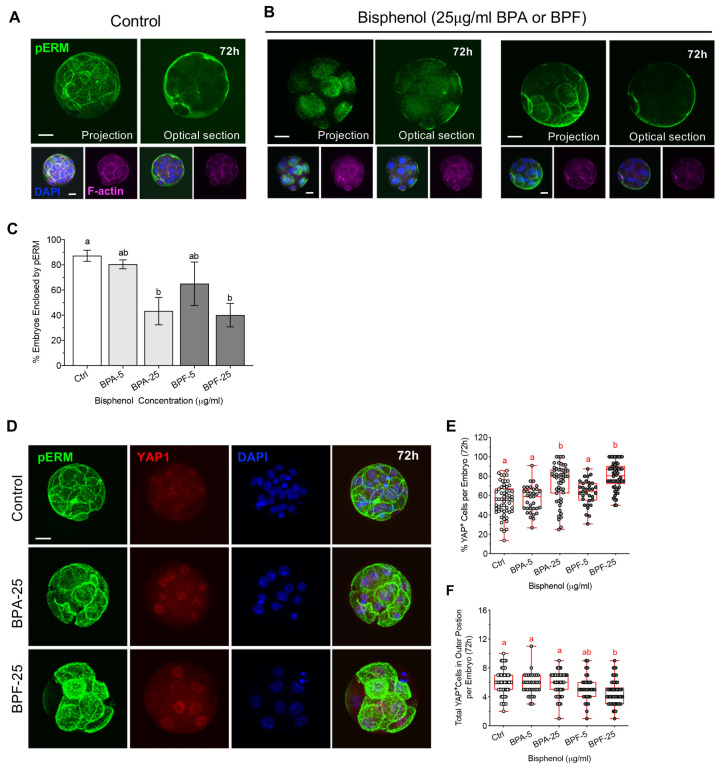
Bisphenol exposure during morula formation disrupts cell polarity and YAP1^+^ cell distribution. (**A**) Representative images (full projection and optical sections) of embryos at 72 hpf from the (**A**) control and (**B**) bisphenol-treated groups, labeled with anti-pERM (green) and F-actin (magenta). DAPI-labeled DNA (blue). Scale bar of 20µm. (**C**) Percent (mean ± s.e.m.) of embryos ‘enclosed’ with pERM labeling over entire surface. Representative data from 3–4 replicates with a total 35–50 embryos in each group. (**D**) Representative images of embryos at 72 hpf, double labeled with anti-pERM (green) and the HIPPO signaling factor, YAP1 (red). DAPI-labeled DNA is shown in blue. (**E**) Percentage of cells per embryo with nuclear YAP1 positive labelling. (**F**) Total cells per embryo located in the outer position with nuclear YAP1 positive labelling. Box and whiskers plots represent the min and max values, with the center horizontal line representing the median. Scale bar of 20 µm. hpf: hours post fertilization. Different letters (red) denote significant differences (*p* < 0.05) between groups.

Considering that actin ring zippering was disrupted in the bisphenol groups, we also assessed cell polarity at the morula stage. Cortical F-actin and pERM labeling of the apical domain in polarized cells were evaluated in embryos exposed to either BPA or BPF from 53–72 hpf relative to controls (Figure 6). In control morula-stage embryos, pERM labeled the apical domain of all blastomeres on the outer surface of the entire embryo. As shown in the optical section, pERM fully covers and encloses the entire embryo surface, indicating polarization of all outer cells as well as increased cell adhesion and compaction (Figure 6A). In contrast, cell polarization was disrupted in embryos exposed to either BPA or BPF. Bisphenol exposed embryos that arrested at the 8-cell stage exhibited pERM staining at individual apical domains that are not connected, while those that reached the morula stage showed disconnected cell–cell interface along the embryo surface or pERM enrichment was restricted to part of the embryo surface, indicating asynchronous/disrupted polarization of the outer cells (Figure 6B). Overall, exposure to high (25 µg/mL) BPA or BPF disrupts cell polarization and significantly reduced the percentage of embryos showing pERM labeling along its entire surface (Figure 6C).

**Figure 7:**
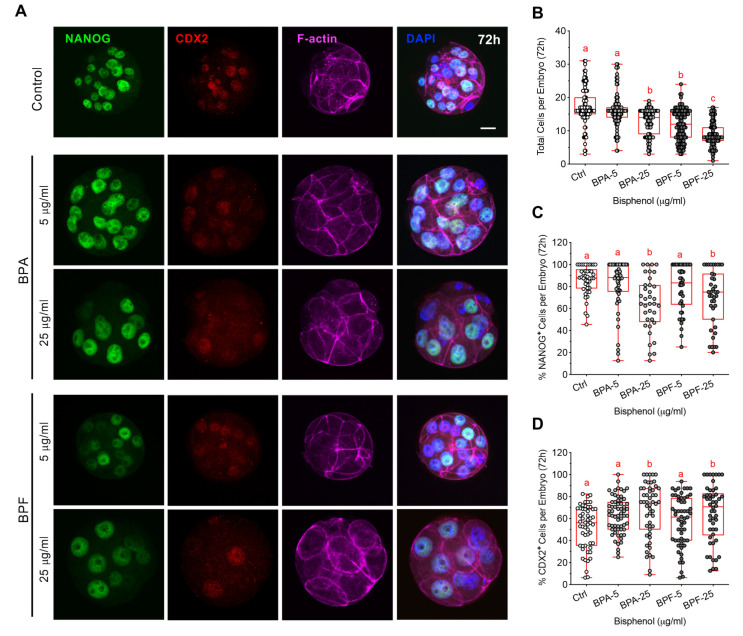
Bisphenol exposure during morula formation disrupts cell division and lineage marker profiles. (**A**) Representative images of embryos at 72 hpf, labelled with F-actin (magenta) together with anti-NANOG (green) and anti-CDX2 (red) to detect inner cell mass (ICM) and trophectoderm (TE) cells, respectively. DAPI-labelled DNA (blue). Scale bar of 20 µm. (**B**) Total cells per embryo in each group at 72 hpf. Percentage of total cells per embryo with (**C**) NANOG and (**D**) CDX2 positive labelling. Box and whiskers plots represent the min and max values, with the center horizontal line representing the median. hpf: hours post fertilization. Different letters (red) denote significant differences (*p* < 0.05) between groups.

Cell polarization has been linked to the regulation of cells fate determination in the embryo. Studies report that in polarized cells, the transcription factor Yes-associated protein 1 (YAP1) promotes expression of CDX2, in the trophectoderm (TE) cells, which are typically located at the peripheral (outer) position in the embryo. Conversely, Hippo signaling suppresses YAP1 and CDX2 expression in unpolarized (apolar) cells that lack the apical domain and are located at a central (inner) position within the embryo [23,27]. Therefore, we assessed whether bisphenol exposure during morula formation affects YAP1 expression and distribution (Figure 6D–F). Interestingly, despite having fewer total cells (Figure 5B), embryos exposed to high dose BPA and BPF contained a significantly higher percent of total cells with YAP1 positive labeling (Figure 6E). Moreover, BPF-exposed embryos showed altered cell positioning (distribution), with fewer total YAP1 positive cells observed at the outer position in the embryo (Figure 6F). This suggests that regulation of cell positioning and/or Hippo signaling, which normally suppresses YAP1 in non-polarized (inner) cells, may be disrupted. Considering the role YAP1 plays in promoting CDX2 expression, next we assessed NANOG and CDX2 labeling (Figure 7A–D) as key markers for the ICM and TE cell lineages, respectively [27,42]. As we previously showed, exposure to BPA or BPF reduced the total cell numbers per embryo (Figure 7B), indicative of impaired cell division. NANOG labeling was detected in the cell nuclei and quantitative analysis revealed that the percent of total NANOG positive cells per embryo was significantly lower following high (25 µg/mL) BPA or BPF exposure (Figure 7C). In turn, consistent with our observed increase in YAP1 positive cells, the percent of total CDX2 positive cells per embryo (Figure 7D) was significantly higher in embryos exposed to BPA and BPF. In sum, these data demonstrate that bisphenol exposure during morula formation impairs cell division and disrupts cell polarization. Additionally, disrupted cell polarization was associated with alterations in the expression and distribution of YAP1 positive cells in these embryos. The proportion of NANOG and CDX2 positive cells was also skewed towards a TE profile in BPA and BPF-exposed embryos, suggestive of disrupted regulation of cell lineage specification.

### 3.6. Bisphenol Exposure during Blastocyst Formation Disrupts Cell Division, Embryo Hatching and Lineage Marker Profiles

**Figure 8:**
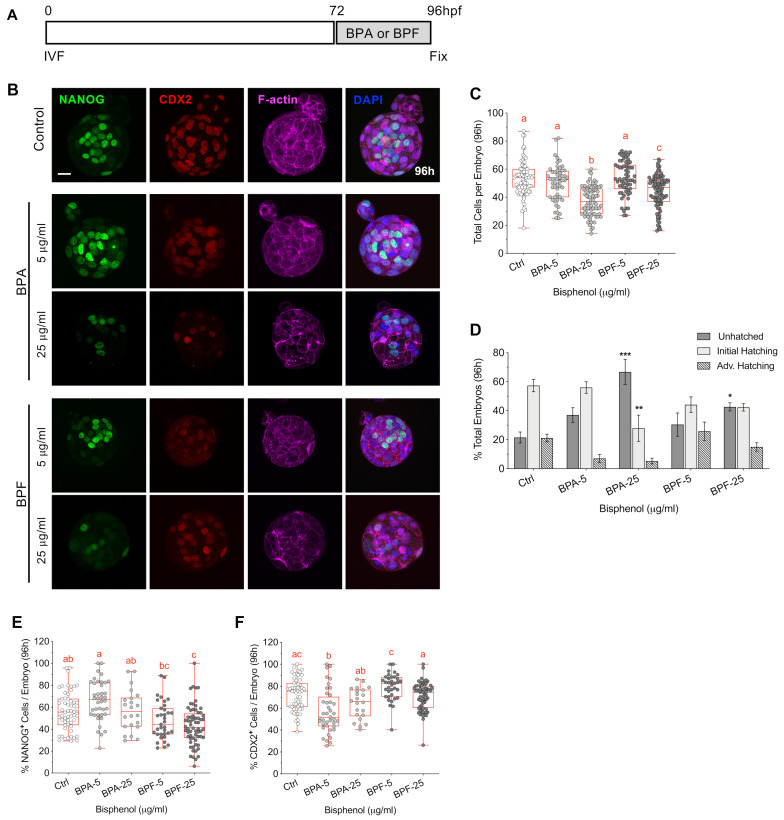
Brief bisphenol exposure during blastocyst formation limits embryo hatching and skews cell lineage specification. (**A**) Timing of BPA or BPF exposure (72–96 hpf, grey) and embryo fixation. (**B**) Representative images of embryos fixed at 96 hpf, labeled with F-actin (magenta) together with anti-NANOG (green) and anti-CDX2 (red) to detect the inner cell mass (ICM) and trophectoderm (TE) cells, respectively. DAPI-labeled DNA (blue). Scale bar of 20 µm. (**C**) Total cells per embryo in each group at 96 hpf. (**D**) Stage of embryo hatching in each group. Percentage of total cells per embryo with (**E**) NANOG and (**F**) CDX2 positive labeling at 96 hpf. Box and whiskers plots represent the min and max values, with the center horizontal line representing the median. hpf: hours post fertilization. Different letters (red) denote significant differences (*p* < 0.05) between groups. * *p* < 0.05, ** *p* < 0.01, *** *p* < 0.001 compared to the control group.

Finally, we tested the impact of bisphenol exposure during development from the morula to blastocyst stage (Figure 8). As previously described, IVF embryos were cultured in KSOM media until 72 hpf then transferred to fresh media (controls) or media supplemented with either BPA or BPF (5, 25 μg/mL) for culture until fixation at 96 hpf (Figure 8A). The embryos were triple labeled to detect F-actin, NANOG and CDX2, while DAPI-labeled DNA was used to assess the number of total cells per embryo (Figure 8B). As we observed in earlier stages tested, embryos exposed to high (25 µg/mL) BPA or BPF contained significantly fewer total cells (Figure 8C), indicative of impaired cell division. Interestingly, culture with BPA during blastocyst formation resulted in fewer total cells per embryo compared to BPF, differing from its less detrimental effect at earlier stages of embryo development. Moreover, exposure to BPA and BPF led to significantly reduced embryo hatching rates compared to controls (Figure 8D), such that the majority (65%) of BPA-treated embryos remained fully enclosed by the zona pellucida with no initiation of hatching.

Immunofluorescence (Figure 8B) analysis also revealed altered cell lineage marker profiles following bisphenol exposure, as reflected by the percentage of NANOG and CDX2 positive cells per embryo (Figure 8E,F). Interestingly, culture with BPA or BPF promoted different responses. Embryos exposed to high (25 µg/mL) BPF contained a lower percentage of NANOG positive cells. Conversely, in BPA exposed embryos, the percentage of NANOG positive cells tended to increase while the percent CDX2 positive cells significantly decreased (Figure 8E,F). These data demonstrate that bisphenol exposure, during blastocyst formation (72–96 hpf), limits cell division and significantly reduces the percentage of embryos that reach the hatching stage. Additionally, cell lineage marker profiles were altered in BPA and BPF-treated embryos. Further studies are needed to assess whether dysregulation of Hippo signaling may play a role in these disrupted profiles.

**Figure 9:**
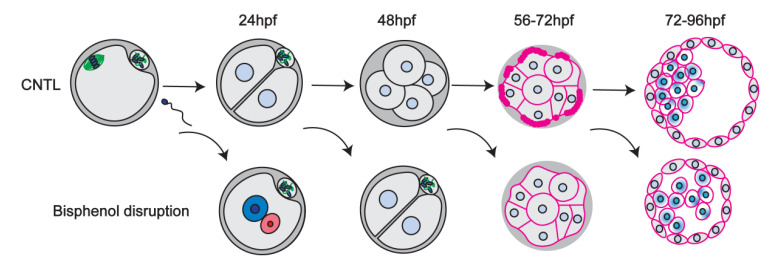
Graphic summary of key transitions during mouse pre-implantation development (first row) and how brief bisphenol exposure (second row) disrupts each stage.

In sum, our comprehensive analysis reveals that brief bisphenol exposure disrupts cell division and cytoskeletal organization that is highly detrimental to pre-implantation embryo development, at all key stages tested (Table 1). A schematic summary (Figure 9) illustrates the disruption of key processes including, cell division, cell polarization, embryo sealing and cell lineage profiles.

## 4. Discussion

In this study we tested the direct effects and underlying mechanisms of bisphenol action during key stages of mouse pre-implantation development. Our results reveal that brief BPA or BPF exposure significantly impedes cell division at each stage assessed. Notably, poor embryo development was associated with disruption of the actomyosin network, cell polarization, embryo sealing at the morula stage, and blastocyst hatching as well as altered cell lineage marker profiles. Surprisingly, BPF exposure was more damaging to the embryo compared to BPA. This comprehensive analysis provides new mechanistic insight into the detrimental effects of bisphenol compounds on cytoskeletal integrity and remodeling that is essential for formation of the early embryo.

Maternal exposure to BPA shortly after fertilization delays embryo development [12,13]. However, the underlying mechanisms by which bisphenols directly affect early embryo formation are poorly understood. Here, we demonstrate that brief culture with either BPA or BPF impairs cell division at all key stages of pre-implantation development, resulting in fewer total cells per embryo. Notably, exposure at the zygote stage (4–24 hpf) disrupted the first cleavage division. Live cell imaging revealed that BPA delays the onset of 2-cell cleavage, while BPF was more damaging such that the majority of zygotes arrested at the pronuclear stage with no mitotic spindle assembly. Bisphenol exposure at the 4–8 cells stage also led to disruption of MT-bridges between the dividing blastomeres that normally connect these cells post division [43], indicative of microtubule defects. Notably, in previous studies we demonstrated that BPA and its common replacement analogue, BPF, exert microtubule disruptive activity in mouse oocytes that perturbs meiotic spindle organization and stability, with BPA being more damaging [29]. Earlier investigations identified tubulin as a BPA target protein [28] and reported disrupted microtubule assembly in plant cells [44] as well as in somatic cell lines cultured with bisphenols [45]. Surprisingly, BPF exposure was more damaging to the early embryo. This differs from our analysis in oocytes [29] and studies in zebrafish embryo-larvae that report higher toxicity for BPA [46]. Culture with BPF resulted in embryos with fewer total cells compared to BPA during most developmental stages we assessed, raising concerns regarding its use as a ‘safe’ replacement analogue. It was only during blastocyst formation when BPA exposure was more detrimental to cell division. This indicates that early embryos show differing levels of susceptibility to the detrimental effects of either BPA or BPF, which may be caused by alternative modes of action and/or depend on the stage of development. Hence, additional studies are warranted to comprehensively investigate the underlying molecular mechanism(s) by which bisphenols disrupt cell division in the embryo.

Importantly, bisphenol exposure during the first cleavage division was detrimental to subsequent embryonic development, despite compound removal. Following transfer to fresh media at 24 hpf, the majority of BPF-exposed embryos arrested after one or two divisions. Only 15% of the embryos developed to the 16-cell stage, underscoring that BPF is highly damaging to the early embryo. In contrast, zygotes exposed to BPA resumed division following compound removal. Yet, analysis at 96 h revealed impaired development. Fewer embryos reached the blastocyst stage and the majority contained significantly fewer total cells. Thus, transient exposure to bisphenols during the zygote stage promotes lasting effects that significantly disrupt subsequent embryonic development. Notably, in addition to cell division defects, bisphenol-exposed embryos were characterized by altered cell lineage marker profiles and pronounced disruption of the actomyosin network. Control embryos showed myosin-II labeling along the actin filament in a well-defined tubular structure beneath the cell membrane. In contrast, bisphenol exposure led to an abnormal punctate F-actin appearance with weak and disconnected myosin-II filaments. These damaging effects on the cytoskeleton can disrupt development, as both cell division and compaction are highly dependent on the force generated by actomyosin contractility [38].

We assessed the impact of bisphenol exposure on the actomyosin network during successive stages of embryo development, beginning with initial cell polarization. At the 8-cell stage, the outer cells of the embryo become polarized with the formation of a distinct apical domain, characterized by an F-actin ring at the cortex and ‘cap-like’ labeling with phospho-ERM (Ezrin/Radixin/Moesin) [10,41]. Our analysis revealed that while apical domain formation ensues in bisphenol-treated embryos, F-actin recruitment and pERM labeling was significantly reduced, and the embryos had fewer polarized cells. Interestingly, apical domain formation ensued in bisphenol-treated embryos that remained arrested at the 4–6 cell stage, thus, demonstrating that regulation of cell polarization is not dependent on blastomere number. Actin plays an important role in apical polarity [39] and phosphorylation of Ezrin is reported to promote F-actin enrichment [41], while de-polymerization of actin abolishes apical domain formation [47]. Moreover, the actomyosin network is essential for the recruitment of polarity proteins including the PAR-aPKC complex to the apical domain [48]. Hence, it will be important to assess the precise mechanisms by which bisphenol exposure can disrupt F-actin and limit apical domain formation.

Cell polarization also plays an important role in embryo sealing, which occurs at the morula stage. This process involves unique F-actin rings as first observed at the 8-cell stage [39]. The actin rings are formed via cortical actin flows together with a polar microtubule network that excludes F-actin from the apical cortex [20]. Elegant studies revealed *de novo* formation of actin rings in outer blastomeres of embryos at the 16-cell stage, which expand and contact the rings on neighboring cells then undergo a ‘zippering’ process that reinforces the tight junctions [20], creating an outer permeability seal that is crucial for blastocele expansion. Notably, brief BPA or BPF exposure during morula formation disrupts embryo sealing, resulting in significantly fewer fully ‘zippered’ embryos. The ‘non-zippered’ embryos were characterized by disconnected myosin-II filaments as well as significantly reduced actin ring expansion and contact between adjacent blastomeres. These results indicate that even brief bisphenol exposure disrupts the establishment and maintenance of the actomyosin network, which was associated with impaired cell polarization as well as embryo sealing. The actomyosin network is important to promote apical domain recruitment and to generate the surface tension to facilitate embryo compaction [17,39]. Consistent with this notion, we observed that BPA or BPF exposure decreased the percentage of embryos with pERM labeling along its entire surface, indicating reduced cell adhesion and compaction at the morula stage. Embryo culture with BPA of BPF during blastocyst formation (72–96 hpf) led to similar disruptions in F-actin labeling. Notably, blastocyst hatching rates were significantly reduced and may be attributable to impaired embryo development, as reflected by lower total cell numbers, as well as incomplete embryo sealing needed for blastocele formation and expansion.

Importantly, exposure to BPA or BPF also altered cell lineage marker profiles in developing embryos. Labeling for the pluripotency marker NANOG together with CDX2 was used to discern the inner cell mass (ICM) and trophectoderm (TE) lineages, respectively [26,27]. Multiple factors regulate ICM and TE cell lineage specification, including cell polarity and the Hippo signaling cascade [23,49,50]. Disruption of the lineage marker profiles was dependent on the timing of bisphenol exposure post fertilization. Transient exposure to low dose BPA at the zygote stage led to blastocysts with an increased percentage of CDX2 positive cells and fewer NANOG positive cells, indicating a skewing towards TE lineage differentiation. Yet, high dose BPA increased the percent NANOG positive cells, suggestive of delayed embryo development and cell differentiation. Previous studies also reported increased NANOG expression in response to BPA exposure in human prostate stem cells [51]. In addition, later exposure to BPA or BPF during morula formation (52–72 hpf) also led to a skewing towards the TE lineage with an increased percentage of CDX2 positive and fewer NANOG positive cells. Interestingly, the percentage of cells labeled with YAP1 that promotes CDX2 expression, also increased. Yet, cell distribution was disrupted in BPF-exposed embryos, with fewer YAP1 positive cells localized in an outer position. In polarized cells, YAP1 binds to TEAD4 and promotes expression on CDX2, in cells typically located at an outer position in the embryo. Conversely, Hippo signaling suppresses YAP1 and CDX2 expression in unpolarized inner cells [23,27]. Hence, the observed altered lineage marker profiles may be associated with disruption of cell polarization and/or Hippo signaling. Finally, embryos exposed to BPA during blastocyst formation (72–96 hpf) showed fewer CDX2 positive cells, which may reflect delayed embryo development and TE cell differentiation as these embryos contained few total cells.

Future studies are needed to determine the underlying mechanisms by which BPA and BPF disrupt cytoskeletal integrity and cell division in the developing embryo. BPA has been shown to target tubulin [28,44] and disrupt microtubule assembly in somatic cell lines, leading to metaphase arrest [45]. Longer term exposure to BPA in somatic cells can also reduce the expression of tubulin and F-actin, as well as cytoskeletal-binding (stabilization) proteins such as cofilin-1, vinculin [52], and cortactin [53]. Moreover, the observed cytoskeletal defects may be linked to bisphenol-mediated mitochondrial damage and/or oxidative stress that has been previously reported in oocytes [54,55,56] and developing embryos [16]. Notably, reactive oxygen species (ROS) can target actin and negatively regulate its polymerization [57]. It will also be important to extend the current analysis using lower bisphenol concentrations. The bisphenol doses we used are similar to previous oocyte [29] and embryo [16] culture studies, but are higher than the range of BPA levels reported in pregnant women, including urine (<0.5–206 ng/mL), serum (0.4–47.1 ng/mL) and placental tissue (<0.5–53 ng/g) samples [1], indicative of chronic environmental exposure. Our aim was to use brief (acute) exposure to test the impact during specific key stages of embryo development, but future studies will assess the response to lower bisphenol levels and maternal exposure shortly after fertilization.

In summary, the current study provides novel insight regarding the direct effects and underlying mechanisms of brief bisphenol exposure during critical stages of pre-implantation embryo development. We show that even brief embryo culture with either BPA or BPF impedes mitotic division at all developmental stages tested. Notably, BPA and BPF disrupts key cytoskeletal components including the actomyosin network that play an important role in regulating cell polarization, actin ring zippering for embryo sealing and cell lineage specification. Finally, our data reveals that BPF is more detrimental to embryonic development compared to BPA, raising concerns regarding its using as a ‘safe’ BPA substitute.

## Figures and Tables

**Table 1:** Summary of Bisphenol Affects During Key Stages of Preimplantation Embryo Development.

Stage of Bisphenol Exposure and Embryo Fixation	Bisphenol-Mediate Disruptions
First Cleavage 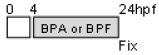	Blocked or delayed onset of 2-cell cleavage BPF more detrimental
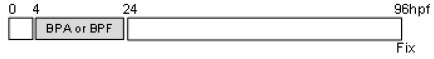	Impaired embryo development Cell division defects Disrupted actomyosin network Altered lineage marker profiles (NANOG, CDX2)
Initial Cell Polarization 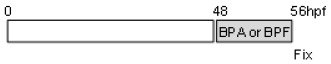	Cell division defects Disrupted cell polarization Fewer polar cells with apical domain
Morula Formation 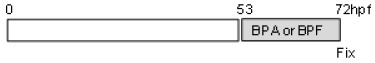	Cell division defects Impaired embryo sealing Disrupted cell polarization & cell position Increased YAP1 positive cells Altered lineage marker profiles (NANOG, CDX2)
Blastocyst Formation 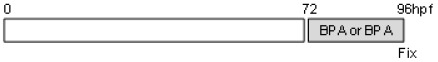	Cell division defects Reduced blastocyst hatching rates Altered lineage marker profiles (NANOG, CDX2)

hpf: hours post fertilization.

## Data Availability

Not applicable.

## References

[1] Lee J., Choi K., Park J., Moon H.B., Choi G., Lee J.J., Suh E., Kim H.J., Eun S.H., Kim G.H. (2018). Bisphenol A distribution in serum, urine, placenta, breast milk, and umbilical cord serum in a birth panel of mother-neonate pairs. Sci. Total Environ..

[2] Ikezuki Y., Tsutsumi O., Takai Y., Kamei Y., Taketani Y. (2002). Determination of bisphenol A concentrations in human biological fluids reveals significant early prenatal exposure. Hum. Reprod..

[3] Dualde P., Pardo O., Corpas-Burgos F., Kuligowski J., Gormaz M., Vento M., Pastor A., Yusà V. (2019). Biomonitoring of bisphenols A, F, S in human milk and probabilistic risk assessment for breastfed infants. Sci. Total Environ..

[4] Vandenberg L.N., Maffini M.V., Sonnenschein C., Rubin B.S., Soto A.M. (2009). Bisphenol-A and the Great Divide: A Review of Controversies in the Field of Endocrine Disruption. Endocr. Rev..

[5] Acconcia F., Pallottini V., Marino M. (2015). Molecular Mechanisms of Action of BPA. Dose Response.

[6] Gore A.C., Chappell V.A., Fenton S.E., Flaws J.A., Nadal A., Prins G.S., Toppari J., Zoeller R.T. (2015). EDC-2: The Endocrine Society’s Second Scientific Statement on Endocrine-Disrupting Chemicals. Endocr. Rev..

[7] Siracusa J.S., Yin L., Measel E., Liang S., Yu X. (2018). Effects of bisphenol A and its analogs on reproductive health: A mini review. Reprod. Toxicol..

[8] Pivonello C., Muscogiuri G., Nardone A., Garifalos F., Provvisiero D.P., Verde N., De Angelis C., Conforti A., Piscopo M., Auriemma R.S. (2020). Bisphenol A: An emerging threat to female fertility. Reprod. Biol. Endocrinol..

[9] Rochester J.R., Bolden A.L. (2015). Bisphenol S and F: A Systematic Review and Comparison of the Hormonal Activity of Bisphenol A Substitutes. Environ. Health Perspect..

[10] Sartain C.V., Hunt P.A. (2016). An old culprit but a new story: Bisphenol A and “NextGen” bisphenols. Fertil. Steril..

[11] Pelch K., Wignall J.A., Goldstone A.E., Ross P.K., Blain R.B., Shapiro A., Holmgren S.D., Hsieh J.-H., Svoboda D., Auerbach S.S. (2019). A scoping review of the health and toxicological activity of bisphenol A (BPA) structural analogues and functional alternatives. Toxicology.

[12] Xiao S., Diao H., Smith M.A., Song X., Ye X. (2011). Preimplantation exposure to bisphenol A (BPA) affects embryo transport, preimplantation embryo development, and uterine receptivity in mice. Reprod. Toxicol..

[13] Pan X., Wang X., Sun Y., Dou Z., Li Z. (2015). Inhibitory effects of preimplantation exposure to bisphenol-A on blastocyst development and implantation. Int. J. Clin. Exp. Med..

[14] Yuan L., Qian L., Qian Y., Liu J., Yang K., Huang Y., Wang C., Li Y., Mu X. (2019). Bisphenol F-Induced Neurotoxicity toward Zebrafish Embryos. Environ. Sci. Technol..

[15] Harnett K.G., Moore L.G., Chin A., Cohen I.C., Lautrup R.R., Schuh S.M. (2021). Teratogenicity and toxicity of the new BPA alternative TMBPF, and BPA, BPS, and BPAF in chick embryonic development. Curr. Res. Toxicol..

[16] Guo J., Zhao M.-H., Shin K.-T., Niu Y.-J., Ahn Y.-D., Kim N.-H., Cui X.-S. (2017). The possible molecular mechanisms of bisphenol A action on porcine early embryonic development. Sci. Rep..

[17] Lim H.Y.G., Plachta N. (2021). Cytoskeletal control of early mammalian development. Nat. Rev. Mol. Cell Biol..

[18] Nikas G., Ao A., Winston R.M., Handyside A.H. (1996). Compaction and Surface Polarity in the Human Embryo in Vitro. Biol. Reprod..

[19] Eckert J.J., Velazquez M.A., Fleming T.P. (2015). Cell Signalling During Blastocyst Morphogenesis. Adv. Exp. Med. Biol..

[20] Zenker J., White M.D., Gasnier M., Alvarez Y.D., Lim H.Y.G., Bissiere S., Biro M., Plachta N. (2018). Expanding Actin Rings Zipper the Mouse Embryo for Blastocyst Formation. Cell.

[21] Tsukita S., Yonemura S. (1999). Cortical Actin Organization: Lessons from ERM (Ezrin/Radixin/Moesin) Proteins. J. Biol. Chem..

[22] Louvet S., Aghion J., Santa-Maria A., Mangeat P., Maro B. (1996). Ezrin Becomes Restricted to Outer Cells Following Asymmetrical Division in the Preimplantation Mouse Embryo. Dev. Biol..

[23] Nishioka N., Inoue K., Adachi K., Kiyonari H., Ota M., Ralston A., Yabuta N., Hirahara S., Stephenson R.O., Ogonuki N. (2009). The Hippo signaling pathway components Lats and Yap pattern Tead4 activity to distinguish mouse trophectoderm from inner cell mass. Dev. Cell.

[24] Handyside A.H. (1980). Distribution of antibody- and lectin-binding sites on dissociated blastomeres from mouse morulae: Evidence for polarization at compaction. J. Embryol. Exp. Morphol..

[25] Johnson M., A Ziomek C. (1981). Induction of polarity in mouse 8-cell blastomeres: Specificity, geometry, and stability. J. Cell Biol..

[26] Strumpf D., Mao C.-A., Yamanaka Y., Ralston A., Chawengsaksophak K., Beck F., Rossant J. (2005). Cdx2 is required for correct cell fate specification and differentiation of trophectoderm in the mouse blastocyst. Development.

[27] Chazaud C., Yamanaka Y. (2016). Lineage specification in the mouse preimplantation embryo. Development.

[28] George O., Bryant B.K., Chinnasamy R., Corona C., Arterburn J.B., Shuster C.B. (2008). Bisphenol A Directly Targets Tubulin to Disrupt Spindle Organization in Embryonic and Somatic Cells. ACS Chem. Biol..

[29] Yang L., Baumann C., De La Fuente R., Viveiros M.M. (2020). Mechanisms underlying disruption of oocyte spindle stability by bisphenol compounds. Reproduction.

[30] Ma W., Baumann C., Viveiros M.M. (2015). Lack of protein kinase C-delta (PKCdelta) disrupts fertilization and embryonic development. Mol. Reprod. Dev..

[31] Machtinger R., Combelles C.M., Missmer S.A., Correia K.F., Williams P., Hauser R., Racowsky C. (2013). Bisphenol-A and human oocyte maturation in vitro. Hum. Reprod..

[32] Nakano K., Nishio M., Kobayashi N., Hiradate Y., Hoshino Y., Sato E., Tanemura K. (2015). Comparison of the effects of BPA and BPAF on oocyte spindle assembly and polar body release in mice. Zygote.

[33] Baumann C., Viveiros M.M. (2015). Meiotic Spindle Assessment in Mouse Oocytes by siRNA-mediated Silencing. J. Vis. Exp..

[34] Santos F., Peters A.H., Otte A.P., Reik W., Dean W. (2005). Dynamic chromatin modifications characterise the first cell cycle in mouse embryos. Dev. Biol..

[35] Wu G., Gentile L., Fuchikami T., Sutter J., Psathaki K., Esteves T.C., Araúzo-Bravo M.J., Ortmeier C., Verberk G., Abe K. (2010). Initiation of trophectoderm lineage specification in mouse embryos is independent of Cdx2. Development.

[36] Komatsu K., Fujimori T. (2015). Multiple phases in regulation of Nanog expression during pre-implantation development. Dev. Growth Differ..

[37] Niwa H., Toyooka Y., Shimosato D., Strumpf D., Takahashi K., Yagi R., Rossant J. (2005). Interaction between Oct3/4 and Cdx2 determines trophectoderm differentiation. Cell.

[38] Maître J.-L., Niwayama R., Turlier H., Nédélec F., Hiiragi T. (2015). Pulsatile cell-autonomous contractility drives compaction in the mouse embryo. Nat. Cell Biol..

[39] Zhu M., Zernicka-Goetz M. (2019). Building an apical domain in the early mouse embryo: Lessons, challenges and perspectives. Curr. Opin. Cell Biol..

[40] Saini D., Yamanaka Y. (2018). Cell Polarity-Dependent Regulation of Cell Allocation and the First Lineage Specification in the Preimplantation Mouse Embryo. Curr. Top. Dev. Biol..

[41] Liu H., Wu Z., Shi X., Li W., Liu C., Wang D., Ye X., Liu L., Na J., Cheng H. (2013). Atypical PKC, regulated by Rho GTPases and Mek/Erk, phosphorylates Ezrin during eight-cell embryocompaction. Dev. Biol..

[42] Marikawa Y., Alarcón V.B. (2009). Establishment of trophectoderm and inner cell mass lineages in the mouse embryo. Mol. Reprod. Dev..

[43] Zenker J., White M.D., Templin R.M., Parton R.G., Thorn-Seshold O., Bissiere S., Plachta N. (2017). A microtubule-organizing center directing intracellular transport in the early mouse embryo. Science.

[44] Adamakis I.-D.S., Panteris E., Eleftheriou E.P. (2019). Tubulin Acetylation Mediates Bisphenol A Effects on the Microtubule Arrays of Allium cepa and Triticum turgidum. Biomolecules.

[45] Pfeiffer E., Rosenberg B., Deuschel S., Metzler M. (1997). Interference with microtubules and induction of micronuclei in vitro by various bisphenols. Mutat. Res..

[46] Moreman J., Lee O., Trznadel M., David A., Kudoh T., Tyler C.R. (2017). Acute Toxicity, Teratogenic, and Estrogenic Effects of Bisphenol A and Its Alternative Replacements Bisphenol S, Bisphenol F, and Bisphenol AF in Zebrafish Embryo-Larvae. Environ. Sci. Technol..

[47] Sun S.C., Wang Q.L., Gao W.W., Xu Y.N., Liu H.L., Cui X.S., Kim N.H. (2013). Actin nucleator Arp2/3 complex is essential for mouse preimplantation embryo development. Reprod. Fertil. Dev..

[48] Zhu M., Leung C.Y., Shahbazi M.N., Zernicka-Goetz M. (2017). Actomyosin polarisation through PLC-PKC triggers symmetry breaking of the mouse embryo. Nat. Commun..

[49] Maître J.-L., Turlier H., Illukkumbura R., Eismann B., Niwayama R., Nédélec F., Hiiragi T. (2016). Asymmetric division of contractile domains couples cell positioning and fate specification. Nature.

[50] Korotkevich E., Niwayama R., Courtois A., Friese S., Berger N., Buchholz F., Hiiragi T. (2017). The Apical Domain Is Required and Sufficient for the First Lineage Segregation in the Mouse Embryo. Dev. Cell.

[51] Prins G.S., Hu W.-Y., Shi G.-B., Hu D.-P., Majumdar S., Li G., Huang K., Nelles J.L., Ho S.-M., Walker C.L. (2014). Bisphenol A Promotes Human Prostate Stem-Progenitor Cell Self-Renewal and Increases In Vivo Carcinogenesis in Human Prostate Epithelium. Endocrinology.

[52] Moreno-Gómez-Toledano R., Arenas M.I., González-Martínez C., Olea-Herrero N., Reventún P., Di Nunzio M., Sánchez-Esteban S., Arilla-Ferreiro E., Saura M., Bosch R.J. (2020). Bisphenol A impaired cell adhesion by altering the expression of adhesion and cytoskeleton proteins on human podocytes. Sci. Rep..

[53] Anahara R., Yoshida M., Toyama Y., Maekawa M., Kai M., Ishino F., Toshimori K., Mori C. (2006). Estrogen agonists, 17.BETA.-estradiol, bisphenol A, and diethylstilbestrol, decrease cortactin expression in the mouse testis. Arch. Histol. Cytol..

[54] Jiao X., Ding Z., Meng F., Zhang X., Wang Y., Chen F., Duan Z., Wu D., Zhang S., Miao Y. (2019). The toxic effects of Fluorene-9-bisphenol on porcine oocyte in vitro maturation. Environ. Toxicol..

[55] Pan M.-H., Wu Y.-K., Liao B.-Y., Zhang H., Li C., Wang J.-L., Hu L.-L., Ma B. (2021). Bisphenol A Exposure Disrupts Organelle Distribution and Functions During Mouse Oocyte Maturation. Front. Cell Dev. Biol..

[56] Nguyen M., Sabry R., Davis O.S., Favetta L.A. (2022). Effects of BPA, BPS, and BPF on Oxidative Stress and Antioxidant Enzyme Expression in Bovine Oocytes and Spermatozoa. Genes.

[57] Balta E., Kramer J., Samstag Y. (2021). Redox Regulation of the Actin Cytoskeleton in Cell Migration and Adhesion: On the Way to a Spatiotemporal View. Front. Cell Dev. Biol..

